# Hardware Implementation and RF High-Fidelity Modeling and Simulation of Compressive Sensing Based 2D Angle-of-Arrival Measurement System for 2–18 GHz Radar Electronic Support Measures

**DOI:** 10.3390/s21206823

**Published:** 2021-10-14

**Authors:** Chen Wu, Denesh Krishnasamy, Janaka Elangage

**Affiliations:** Defence Research and Development Canada-Ottawa Research Centre, Ottawa, ON K1A 0Z4, Canada; Denesh.krishnasamy@forces.gc.ca (D.K.); Janaka.elangage@forces.gc.ca (J.E.)

**Keywords:** angle of arrival, random spaced array, ultra-wideband digital receiver, compressive sensing, RF high-fidelity modeling and simulation, radar electronic support measures, radar electronic warfare

## Abstract

This article presents the hardware implementation and a behavioral model-based RF system modeling and simulation (M&S) study of compressive sensing (CS) based 2D angle-of-arrival (AoA) measurement system for 2–18 GHz radar electronic support measures (RESM). A 6-channel ultra-wideband RF digital receiver was first developed using a PXIe-based multi-channel digital receiver paired with a 6-element random-spaced 2D cavity-backed-spiral-antenna array. Then the system was tested in an open lab environment. The measurement results showed that the system can measure AoA of impinging signals from 2–18 (GHz) with overall RMSE of estimation at 3.60, 2.74, 1.16, 0.67 and 0.56 (deg) in L, S, C, X and Ku bands, respectively. After that, using the RF high-fidelity M&S (RF HF-M&S) approach, a 6-channel AoA measurement system behavioral model was also developed and studied using a radar electronic warfare (REW) engagement scenario. The simulation result showed that the airborne AoA measurement system could successfully measure an S-band ground-based target acquisition radar signal in the dynamic REW environment. Using the RF HF-M&S model, the applicability of the system in other frequencies within 2–18 (GHz) was also studied. The simulation results demonstrated that the airborne AoA measurement system can be used for 2–18 GHz RESM applications.

## 1. Introduction

The angle-of-arrival (AoA) of the signal of interest (SOI) is the most important and expensive measurement parameter in any radar electronic support measures (RESM) system to de-interleave intercepted signals [1,2,3,4] since the frequency agility technology has been widely used in modern military radar systems, and multiple RF/microwave receiving channels are often needed in an AoA measurement system. Traditionally, spinning direction-finding (DF) antenna, amplitude-comparison, phase-comparison, interferometry [5,6,7], and time difference of arrival methods are popular AoA measurement approaches used in RESM systems [8,9,10,11,12]. In addition, there are many RF/microwave DF systems developed for wireless communication applications. Tuncer et al. [13] give a good discussion of these systems. From signal processing perspective, there are a number of algorithms used for signal DF in both military and commercial applications. Among them, multiple signal classification (MUSIC) [14] and estimation of signal parameters via rotation invariance techniques (ESPIRT) [15] are commonly used. However, to apply these methods, generally, a stable signal environment is required since the measured signal covariance matrix needs to be used in these algorithms. Many other AoA estimators have also been developed, focusing on different array structures and fast processing speeds. Some examples of such developments were given in [16,17,18,19,20,21,22,23]. Lonkeng et al. [24] presented the research work on 2D DF estimation using arbitrary arrays in MIMO systems and introduced the 2D Fourier domain line search MUSIC algorithm. Recently, based on information geometry (IG), Dong et al. [25] introduced a simple scaling transform-based information geometry method, which had more consistent performance than the original IG method with higher AoA estimation resolution.

To estimate SOI AoA in a non-stationary environment, the Direct Data Domain (D3) method was introduced in [26,27,28,29,30], and it was applied to 2D AoA measurement for RESM application. Wu et al. [31] introduced a D3-based 2D AoA estimator operating from 6 to 18 GHz and focused on measuring the low probability of intercept radar signals [1,32]. The approach can measure the SOI AoA with just two complex snapshots from a 2D 7-element nonuniformly spaced array in high SNR scenarios. Using more snapshots (e.g., 1024 samples), it can estimate intercepted signal’s AoA in low SNR scenarios. In [31], commonly used radar signals were studied, including Barker code of length 13, two-value frequency-coded waveform, poly-phase waveform, and frequency-modulated continuous wave signal.

The compressive sensing (CS) framework [33,34,35] has been used in many areas. Refs. [36,37] give some examples. Recently, Wu et al. [38,39] introduced a new multi-emitter 2D AoA estimator from 2–18 GHz based on signal spatial-sparsity [40] and Dantzig Selector [41] methods. The key features of the estimator are no requirement of a priori knowledge of intercepted signals, including signal frequencies, and its capability of processing multiple simultaneous incoming signals within the 2D array field-of-view (FOV) and the instantaneous bandwidth of microwave digital receivers. A good summary of the current CS-based AoA methods, including Bayesian CS for direction-of-arrival estimation [42,43], is also given in [39].

To develop the CS-based AoA measurement system introduced in [39] with the considerations of using the system in RESM for radar electronic warfare (REW) applications, this article presents the research results of (1) the AoA estimation system hardware implementation, (2) the open lab-based AoA measurement setup, (3) the system-level RF high-fidelity modeling and simulation (RF HF-M&S), and (4) measurement and simulation results. More specifically, the article presents the following in detail:

- A 6-channel 2–18 GHz RF/digital receiver hardware, using PXIe form-factor, integrated with a 6-element 2–18 GHz cavity-backed-spiral-antenna (CBSA) array with randomly located element positions given in [39];
- To demonstrate that the CS-based AoA method introduced in [39] can be used in dynamic REW engagement environment, the RF HF-M&S approach was used to model:
  ○ An engagement scenario in the System Tool Kit (STK) [44] with a ground-based S-band target acquisition radar (TAR) and an aircraft equipped with a RESM system that has the CS-based AoA measurement capability;
  The S-band radar transmitting system behavioral model in SystemVue (SVE) [45];
  A 6-channel microwave-digital receiving system behavioral model in SVE, and
  The CS-based AoA algorithm [39] in Matlab.
- The measurement setup for AoA lab test from 2 to 18 GHz, and
- Measurement and simulation results.

The main contributions of this article are: this is the first demonstration of the novel CS-based AoA estimation scheme used in the REW scenario through the RF HF-M&S, and the hardware implementation and measurements that validate the scheme can produce correct AoA estimations from 2 to 18 GHz. The results presented in this article prove that the theory developed in [39] can be applied in RESM application, and the system can produce good quality AoA estimations in an ultra-wide frequency band.

The remainder of this paper is organized as follows. Next section briefly outlines the CS-based 2D AoA algorithm using randomly-spaced 2D antenna arrays. The details of the method and its studies can be found in [39]. A 6-channel PXIe-based ultra-wideband RF receiver and antenna array hardware and the AoA open lab test setup are presented in Section 3. In Section 4, using the RF HF-M&S approach, a REW engagement scenario modeled in the STK is first discussed, and then the top-level of the RF system behavioral model in SEV is presented. The measurement and M&S results are in Section 5. Section 6 has the conclusions. Appendix A has a brief introduction of the RF HF-M&S method, and Appendix B gives the detailed behavioral models and RF performance of the TAR transmitter and the 6-channel RESM receiver modeled in the SVE. Appendix C has the acronym list.

## 2. CS-Based 2D AoA Algorithm Using Randomly-Spaced Array (RSA)

### 2.1. Outline of the Method

**Figure 1:**
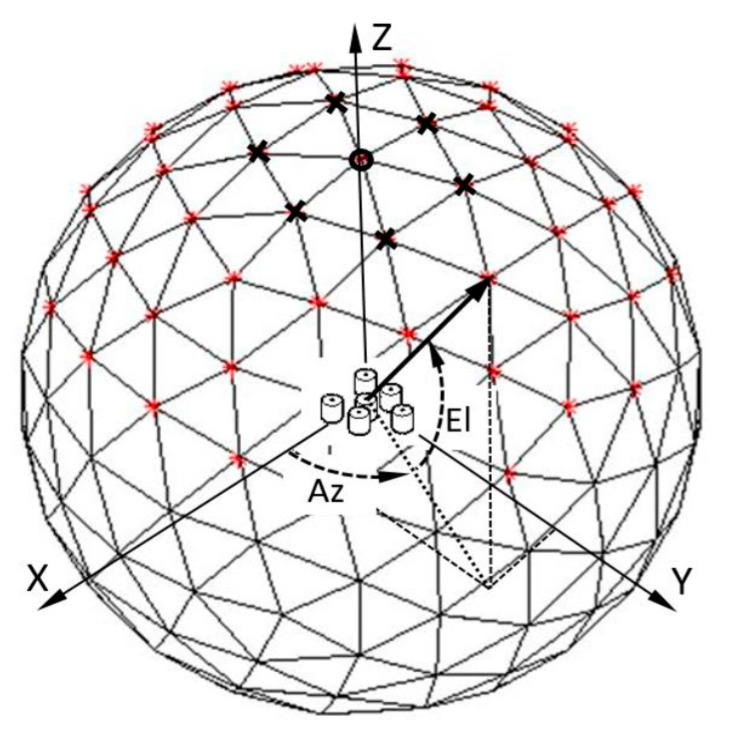
An illustration of a 2D-angle-grid formed by an icosphere-based mesh, and a 6-element RSA. The array phase-reference center (defined at the first element in the center of the array) and the icosphere center are collocated at the origin of the XYZ-coordinate system [39].

Let us consider a 2D RSA with a 2D-angle-grid defined as in Figure 1. The 2D-angle-grid is defined by the icosphere structure [46], which is formed by subdividing the triangles of a regular icosahedron, and the number of subdivisions (W) determines the density of the vertices on the surface of an icosphere [47]. The line between the origin and one of the vertices defines an AoA direction and is represented by azimuth (Az) and elevation (El) angles. The mesh’s angular resolution level can be determined by averaging El-angle differences between the vertex on the z-axis (marked by a circle) and surrounding 6 vertices (marked by cross). For example, the resolution is 0.54 degrees, when W is equal to 7, and there are total of 29,495 vertices that are close to evenly distributed on 4π solid angle. Suppose the array FOV is defined within [${\mathrm{Az}}_{1\;}{\mathrm{Az}}_{2}]\times \left[{\mathrm{El}}_{1}{\;\mathrm{El}}_{2}\right]$ and it is discretized into a 2D-angle-grid in front of the RSA. For example, the red vertices in Figure 1 are inside the array FOV.

Within [${\mathrm{Az}}_{1\;}{\mathrm{Az}}_{2}]\times \left[{\mathrm{El}}_{1}{\;\mathrm{El}}_{2}\right]$, there are total N close to equally spaced vertices, and each vertex defines a direction $\left({\mathrm{az}}_{n},{\mathrm{el}}_{n}\right)\;$(n=1, 2, 3, …N), on which the CS dictionary Ψ matrix is defined. If there are S non-coherent signals from $\left({\mathrm{az}}_{s},{\mathrm{el}}_{s}\right)\;$(s=1, 2, 3, …S≪N) directions received by the array, this forms an S-sparse problem and can be expressed as Ψx, and$\;x={\left[0\cdots 1\cdots 0\;1\cdots 0\right]}^{T},{\left\|x\right\|}_{0}=S$. T is non-conjugate transposition. In order to find nonzero locations in x, K (≪N) measurements have to be performed in the CS framework. In our AoA measurement system, a 2–18 GHz CBSA array with K randomly located elements provides the measurement data y. Equation (1) gives the relation between x and y.

(1) y = Ax

where A=ΦΨ is the CS recovery matrix, in which  Φ  is known as the CS sensing matrix. Then the problem can be solved by using the Dantzig selector to find those nonzero locations in  x as described in [38,39].

In the rest of the section, we highlight how to apply the AoA method to wideband RESM applications without a priori knowledge of incoming signal frequencies using the 2–18 GHz CBSAs and ultra-wideband digital receivers.

### 2.2. Measured Data from K Digital Receivers Connected to A K-Element RSA

It was assumed that the sampled time-domain signal voltage from the kth digital receiver is

(2) $$v_{k}=\left[v_{k}\left(1\right){\;v}_{k}\left(2\right)\;\cdots {\;v}_{k}\left(m\right)\;\cdots {\;v}_{k}\left(N_{\mathrm{FFT}}\right)\right]\;$$

where m indicates the mth time-step. After $N_{\mathrm{FFT}}$-point FFT on the measured time-domain data, we have K-set of frequency-domain data. If there are S local-peaks higher than a pre-defined detection-threshold level in the frequency-domain data obtained from the first element in the center of the RSA, then we can have a set of measured frequency-domain complex data$\;u_{k}\left(s\right)$, where k=1, 2,⋯,K and  s=1, 2, ⋯, S. We also have S estimated frequencies$\;f_{e}\left(s\right)$ from FFT. Thus the measured data at each estimated frequency is

(3) $$M\left(s\right)={\left[1\;\frac{u_{2}\left(s\right)}{u_{1}\left(s\right)}\;\cdots \;\frac{\;u_{k}\left(s\right)}{u_{1}\left(s\right)}\;\cdots \;\frac{u_{K}\left(s\right)}{u_{1}\left(s\right)}\right]}^{T}$$

Here, the intercepted signal sources are in far-field of the RSA, and the mutual coupling between CBSA elements is negligible [48]. Note that (3) is the measured array steering vector (ASV) in the sth direction, and the phase references the first (center) element.

### 2.3. Dictionary, Sensing and Recover Matrices

Using $\left({\mathrm{az}}_{n},{\mathrm{el}}_{n}\right)\;$(n=1, 2, 3, …N), the dictionary matrix in our problem is defined as

(4) $$\Psi ={\left[\begin{array}{ccc} \begin{array}{c} k_{x}\left(1\right)\\ k_{y}\left(1\right)\\ k_{z}\left(1\right) \end{array} & \begin{array}{cc} \cdots & \begin{array}{c} k_{x}\left(n\right)\\ k_{y}\left(n\right)\\ k_{z}\left(n\right) \end{array} \end{array} & \begin{array}{cc} \cdots & \begin{array}{c} k_{x}\left(N\right)\\ k_{y}\left(N\right)\\ k_{z}\left(N\right) \end{array} \end{array} \end{array}\right]}_{3\times N}$$

where

(5) $$\left[\begin{array}{c} k_{x}\left(n\right)\\ k_{y}\left(n\right)\\ k_{z}\left(n\right) \end{array}\right]=\left[\begin{array}{c} \cos\left({\mathrm{el}}_{n}\right)\cos\left({\mathrm{az}}_{n}\right)\\ \cos\left({\mathrm{el}}_{n}\right)\;\sin\left({\mathrm{az}}_{n}\right)\\ \;\sin({\mathrm{el}}_{n})\; \end{array}\right]$$

note that each column in (4) has a unit norm, and the nth column is the normalized-wave-vector in nth direction.

The random element locations define the sensing matrix

(6) $$\Phi ={\left[\begin{array}{c} \begin{array}{ccc} x_{1} & y_{1} & z_{1}\\ x_{2} & y_{2} & z_{2} \end{array}\\ \vdots \\ \begin{array}{c} \begin{array}{ccc} x_{k} & y_{k} & z_{k} \end{array}\\ \vdots \\ \begin{array}{ccc} x_{K} & y_{K} & z_{K} \end{array} \end{array} \end{array}\right]}_{\;K\times 3}$$

and it was assumed${\;z}_{k}=0$, and$\;\left[x_{1}\;y_{1}\;z_{1}\right]=\left[0\;0\;0\right]$ in following discussion. The location samples in Φ are independent and identically distributed. Then the recovery matrix (A) from (4) and (6) is

(7) $$A={\left[\begin{array}{ccccc} x_{1}k_{x}\left(1\right)+y_{1}k_{y}\left(1\right) & \cdots & x_{1}k_{x}\left(n\right)+y_{1}k_{y}\left(n\right) & \cdots & x_{1}k_{x}\left(N\right)+y_{1}k_{y}\left(N\right)\\ \vdots & & \vdots & & \vdots \\ x_{k}k_{x}\left(1\right)+y_{k}k_{y}\left(1\right) & \cdots & x_{k}k_{x}\left(n\right)+y_{k}k_{y}\left(n\right) & \cdots & x_{k}k_{x}\left(N\right)+y_{k}k_{y}\left(N\right)\\ \vdots & & \vdots & & \vdots \\ x_{K}k_{x}\left(1\right)+y_{K}k_{y}\left(1\right) & \cdots & x_{K}k_{x}\left(n\right)+y_{K}k_{y}\left(n\right) & \cdots & x_{K}k_{x}\left(N\right)+y_{K}k_{y}\left(N\right) \end{array}\right]}_{\;K\times N}$$

if there is only one signal from s^th^ direction, we have

(8) $$Ax_{s}=A\left(s\right)={\left[\begin{array}{c} \begin{array}{c} x_{1}\cos\left({\mathrm{el}}_{s}\right)\cos\left({\mathrm{az}}_{s}\right)+{\;y}_{1}\cos\left({\mathrm{el}}_{s}\right)\sin\left({\mathrm{az}}_{s}\right)\\ \vdots \end{array}\\ \begin{array}{c} x_{k}\cos\left({\mathrm{el}}_{s}\right)\cos\left({\mathrm{az}}_{s}\right)+{\;y}_{k}\cos\left({\mathrm{el}}_{s}\right)\sin\left({\mathrm{az}}_{s}\right)\\ \vdots \end{array}\\ x_{K}\cos\left({\mathrm{el}}_{s}\right)\cos\left({\mathrm{az}}_{s}\right)+{\;y}_{K}\cos\left({\mathrm{el}}_{s}\right)\sin\left({\mathrm{az}}_{s}\right) \end{array}\right]}_{\;K\times 1}$$

where$\;x_{s}={\left[0\;\cdots 1\cdots 0\right]}^{T}$, i.e., only the sth element is nonzero. It was also assumed that the sth signal direction is on one of the vertices of the 2D-angle-grid, which is called the on-grid case. Source signal direction off the 2D-angle-grid is called the off-grid case. Test signals from both cases are used in the study.

Moreover, based on ASV definition and considering (8), we have

(9) $$A\left(s\right)=\frac{\lambda }{j2\pi }\ln(ASV\left(s\right))$$

where λ is the free-space wavelength.

### 2.4. Equations Used for Recovering $x_{s}$

Since all the concurrent signals are non-coherent signals and digital receivers operate at their linear conditions, the multiple-signal AoA estimation problem can be decomposed into S equations in (10), using measured data in (3).

(10) $$\frac{\lambda_{e}\left(s\right)}{j2\pi }\ln(M\left(s\right))=Ax_{s}+\eta ,\;\left(s=1,\;2,\;3,\cdots S\right)$$

$\lambda_{e}\left(s\right)=c/{\;f}_{e}\left(s\right)$ is the estimated free-space wavelength, η  is the receiver noise, and c is the light speed in free space. Then (10) is solved by the Dantzig selector.

For the off-grid case, our method just picks the location of the maximum number for each$\;x_{s}$ obtained by the Dantzig selector. The Matlab code developed in [39] was used in this study, and the Dantzig selector solver was obtained from [49].

## 3. Hardware Implementation of the 6-Channel RESM Digital Receiver and Open Lab Measurement Setup

### 3.1. Randomly-Spaced CBSA Array

Ref. [39] studied a number RSA structures. Based on the system cost and AoA measurement performance, it suggested using the 6-element array. Note that the study in [39] also suggested that the element locations of element-2 to -6 can be randomly picked on the aluminum disk, as long as they do not bias too much in one area.

**Figure 2:**
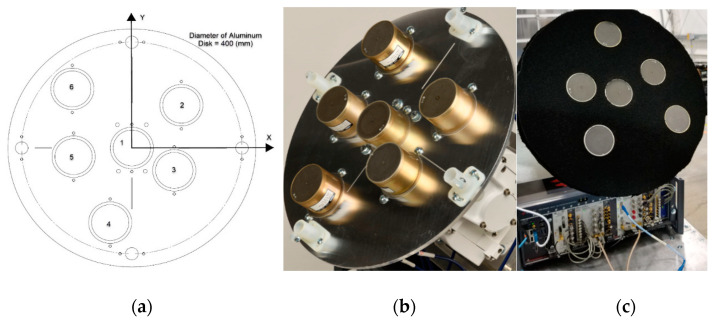
(**a**) 6-element 2–18 GHz CBSA array, array hardware (**b**) without and (**c**) with absorbing material.

A drawing of a 6-element array using 2–18 GHz CBSA is shown in Figure 2a, and its hardware is shown in Figure 2b. The locations of the elements in this study are given in Table 1. To reduce the reflection from the aluminate plate, microwave absorbing material was inserted between elements, as shown in Figure 2c. The details of CBSAs can be found in [50].

### 3.2. PXIe Ultra-Wideband Microwave Digital Receiver

**Figure 3:**
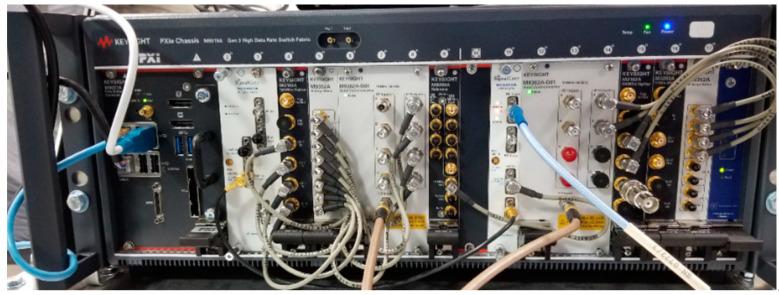
PXIe 8-channel microwave digital receiver.

**Figure 4:**
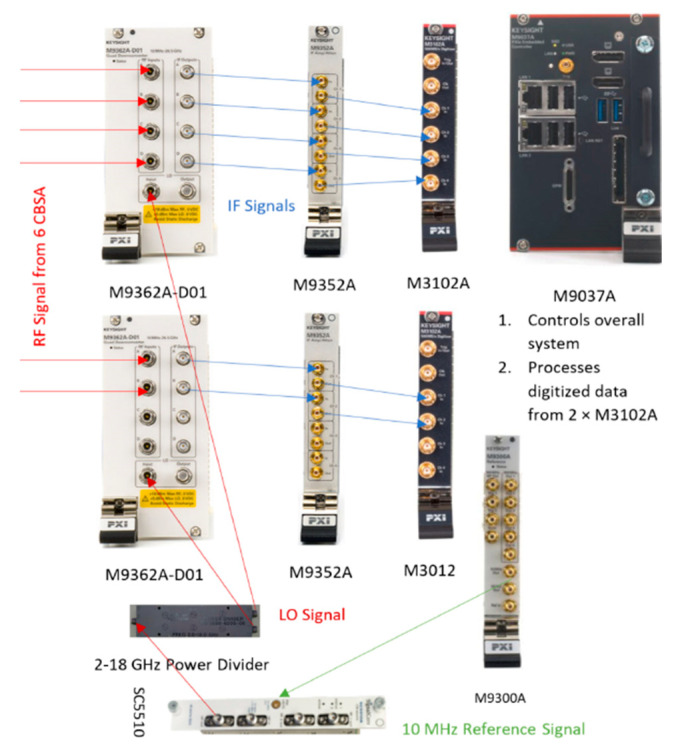
Signal paths in the PXIe receiving system in Figure 3.

The PXIe based 8-channel microwave receiver hardware assembled in an 18-slot Keysight PXIe M9019A Gen-3 chassis is shown in Figure 3. Six of the eight channels were connected to the CBSA array to form the RESM receiver. The PXIe modules used in the receiver are listed in Table 2, with their slot locations in the chassis. The first five modules were from Keysight Technologies, Inc., and the last module was from SignalCore. The signal flow from RF to IF, and then sampling and processing are shown in Figure 4.

In the receiver, the RF signal down-converted to 100 MHz and sampled at 500 Meg-sample/s. 4096-point Fast Fourier Transform (FFT) was used to process the real data in the CS-based AoA estimation algorithm. The detail of the algorithm was discussed in [39].

### 3.3. The Open Lab AoA Measurement Setup

**Figure 5:**
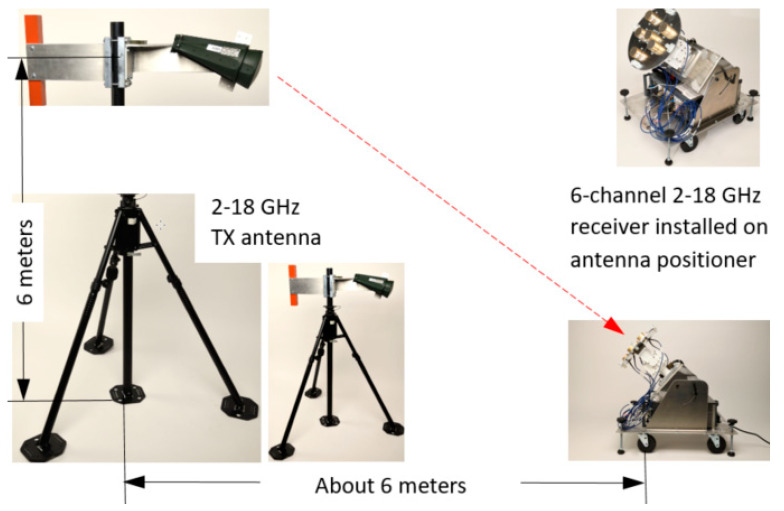
The TX and RX array assemblies for an open lab test: the measurement setup was in an open area, and the microwave absorbing materials were placed in front of the antenna positioner to absorb ground reflections.

The CBSA array and multi-channel receiver were integrated together, as shown in Figure 5. In the figure, the array was installed on an antenna positioner, which is an azimuth-on-elevation positioner system, and connected to 6 channels of the PXIe digital receiver. The receiving array was placed in the far-field of the TX antenna that was a 2–18 GHz dual-ridge dual-linear polarization circular horn antenna that has the same performance as the antenna in [51]. In the test, only a horizontal polarized signal was used. Microwave absorbing materials were also placed (not shown in the figure) on the ground in front of the antenna positioner.

Before the measurement, the height of the TX antenna was selected by adjusting the antenna mast, thus that the incident angle was at El =90 (deg). Then by rotating the antenna positioner angles, the incident Az-angle of the TX-signal was changed and recorded.

The tested incident angles were:

- Az from −180 to 180 (deg) at 30 (deg) interval, and
- El from 30 to 90 (deg) at 10 (deg) interval.

**Figure 6:**
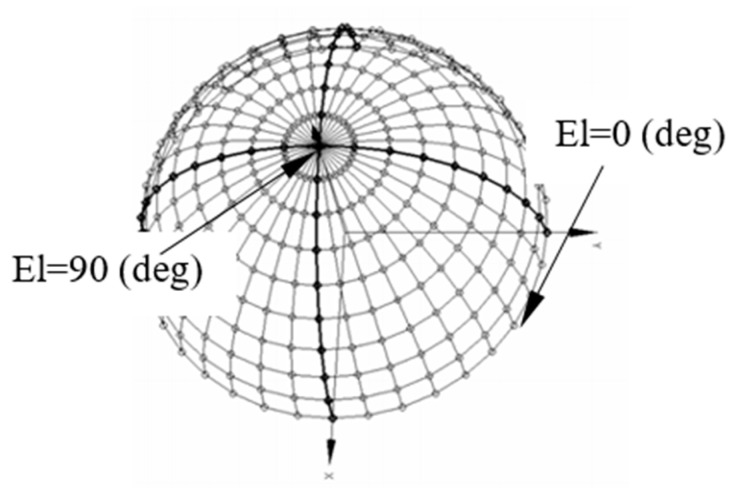
An example of defining the directions on the UV-sphere.

Thus, there were a total of 13 and 7 angles measured for Az and El, respectively, i.e., at each frequency, there were 91 directions. During the measurement, since the positioner was an azimuth-on-elevation system, we set an El-angle first and then rotated the Az-angle. At each Az-angle, the frequency was scanned from 2 to 18 (GHz) at 1 (GHz) interval. The Az- and El-angles define the directions in the UV-sphere, as illustrated in Figure 6.

The received RF signal was down-converted to IF frequency, sampled by 14-bit high-speed digitizer at 500 (Mega-Sample/s), and then the sampled data were processed by 4096-point real data FFT. At each incident angle (El, Az), using the first channel FFT data, the frequency $\left(f_{\mathrm{IF}}\right)$ that had the peak FFT value was found. Using this${\;f}_{\mathrm{IF}}$, a set of 6 complex numbers $\left(M_{i}\left(\mathrm{El},\;\mathrm{Az},f_{\mathrm{IF}}\right),\;i=1,\ldots 6\right)$ can be obtained from the 6 channels. The normalized data were ${\;m}_{i}\left(\mathrm{El},\;\mathrm{Az},f_{\mathrm{IF}}\right)={\;M}_{i}\left(\mathrm{El},\;\mathrm{Az},f_{\mathrm{IF}}\right)\;/M_{1}\left(\mathrm{El},\;\mathrm{Az},f_{\mathrm{IF}}\right),$ where $M_{1}\left(\mathrm{El},\mathrm{Az},f_{\mathrm{IF}}\right)\;$ was the data from the first channel.

Note that, since the 6 CBSAs were not phase-matched elements and the RF receivers were not identical, at each frequency, the 13 measured data at El=90 (deg) were used as the calibration data to remove the amplitude and phase discrepancies caused by the CBSAs and RF digital receivers. Therefore, at each frequency measurement point, the data sent to CS-based AoA algorithm was ${\;m}_{i}\left(\mathrm{El},\;\mathrm{Az},f_{\mathrm{IF}}\right)/m_{i}\left(90,\;\mathrm{Az},f_{\mathrm{IF}}\right)$. The frequency used to estimate AoA is ${\;f}_{\mathrm{LO}}+f_{\mathrm{IF}}$, in which $f_{\mathrm{LO}}$ was receiver local oscillator frequency. As mentioned earlier, this is one of the advantages of the CS-based AoA method that it does not need to know the intercepted signal frequency, and the frequency used for AoA calculations was measured by the receiver itself. Hence, the method can be used in an ultra-wide frequency band. The operational frequency band was determined by the antenna elements and receiver hardware.

## 4. Using RF HF-M&S Methodology to Study CS-Based AoA Estimator in REW Environment

This section presents a vignette that demonstrates the use of the CS-base 2D AoA measurement sensor in REW scenario in the RF HF-M&S. The vignette had a 6-channel airborne RESM receiver intercepted a ground-based TAR signal, and the receiver estimated the direction of the radar signal. A brief introduction of the RF HF-M&S methodology can be found in Appendix A.

### 4.1. The Vignette of the TAR Signal AoA Measurement by the Airborne CS-Based AoA Estimator

**Figure 7:**
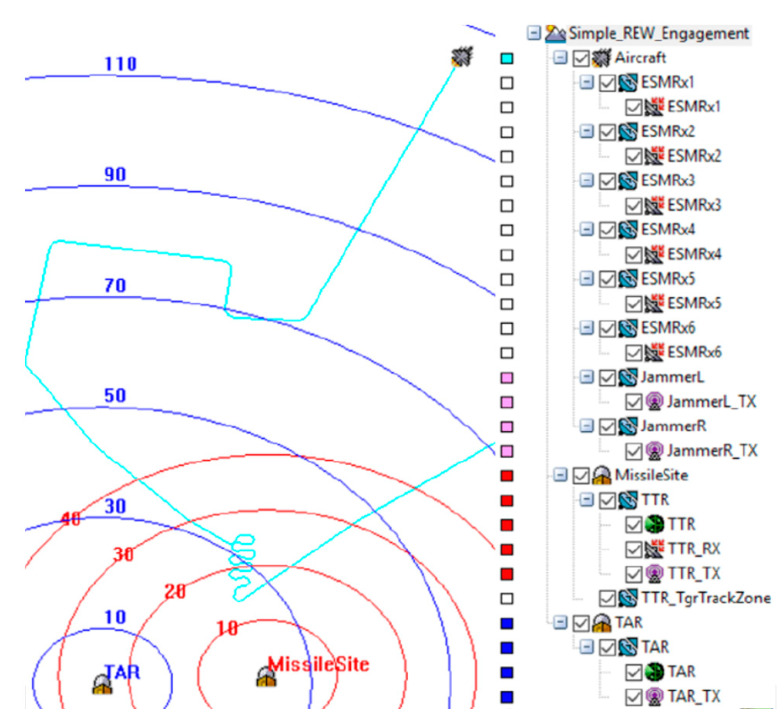
Top view of the beginning of the simple REW engagement scenario, the blue and red circles indicate the ranges from radar centers in kilometers and the hierarchy of STK models (TAR: target acquisition radar, TTR: target tracking radar).

Figure 7 shows the top view of a REW engagement scenario. An aircraft installed with a multi-channel RESM sensor (ESMRxi (i = 1 to 6)) and a jammer system was approaching an area defended by an RF guided missile system, which was modeled by TAR and MissileSite. The cyan-line shows the aircraft flight path projected on the ground. In the figure, the aircraft icon was located at the beginning of the scenario. The vignette of the scenario to be used to demonstrate the multi-channel CS-based AoA measurement system was that the RESM receiver intercepts the TAR signal whenever the RESM receiving antennas intercept the TAR signal during the flight, the signal’s AoA is measured by the RESM system. The constraints that define the RESM receiver can intercept the TAR signal will be discussed later.

**Figure 8:**
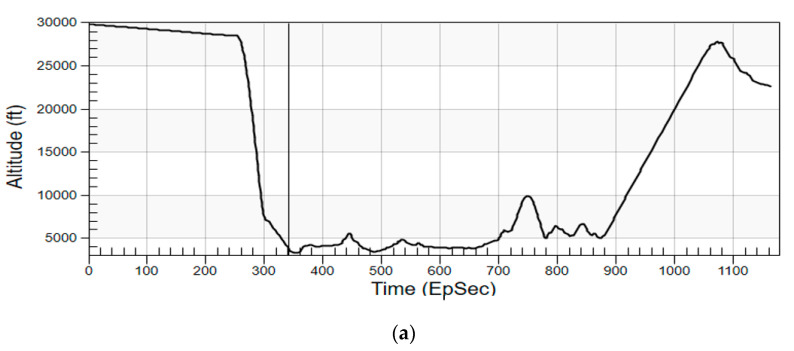

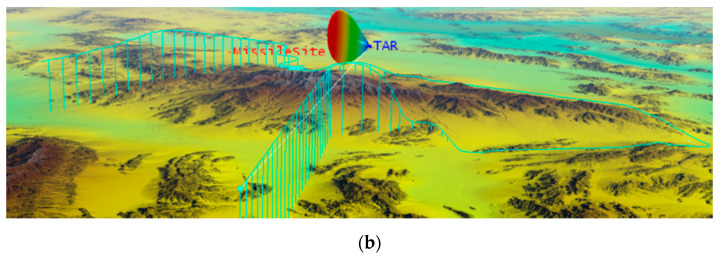
(**a**) Altitude of the aircraft in the scenario, (**b**) cyan drop-lines indicates the aircraft from terrain surface at different times (EpSec: Epoch Second).

The aircraft altitude in the scenario is plotted in Figure 8a. The vertical cyan lines in Figure 8b show the distances between the aircraft and terrain surface at different moments. One can see that after the onboard RESM detects and measures TAR signal, the aircraft dramatically reduces its altitude and flies in between mountains. The dynamically changing aircraft flight trajectory and attitude form the first constraint in the RF HF-M&S. The RESM receiving antennas can only intercept the TAR signal when the line-of-sight (LOS) condition is met.

**Figure 9:**
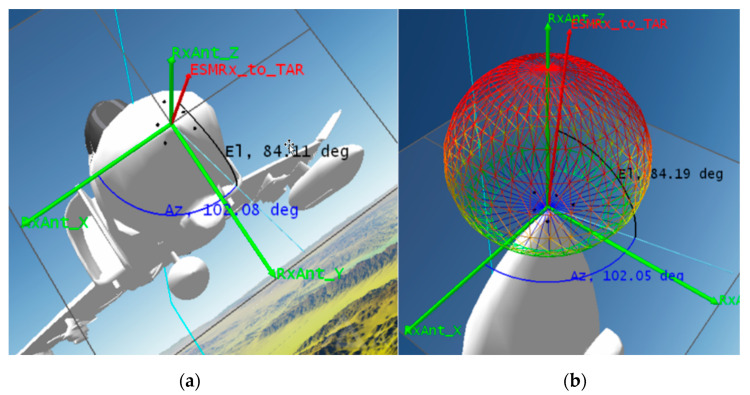
(**a**) 6-element RESM RX array and array local coordinate system, (**b**) an example of the first CBSA element radiation pattern.

Figure 9a shows the 6-element RESM receiving array installed at the nose of the aircraft. The origin of the array local coordinate system was located at the phase center of the first element, and other element phase center locations were indicated by the black dots in the figure, and Table 1 gives their coordinates. Figure 9 also shows the current direction from the array to the TAR by the red vector called ESMRX_to_TAR. This vector was defined by Az and El angles in blue and black lines, respectively, in the array’s local coordinate system RxAnt_(x, y, z). The center element radiation pattern is shown in Figure 9b, which has about 100 (deg) 3-dB tbeamwidth the at S-band. Other elements have the same antenna pattern and are not shown in the figure. The second constraint used in the M&S was that the CS-based AoA algorithm only processes signals within the antenna’s 3-dB beamwidth. Using this constraint together with LOS constraint discussed earlier, STK generates the time slots (TS) of RESM antenna accessing the TAR signal as listed in Table 3.

**Figure 10:**
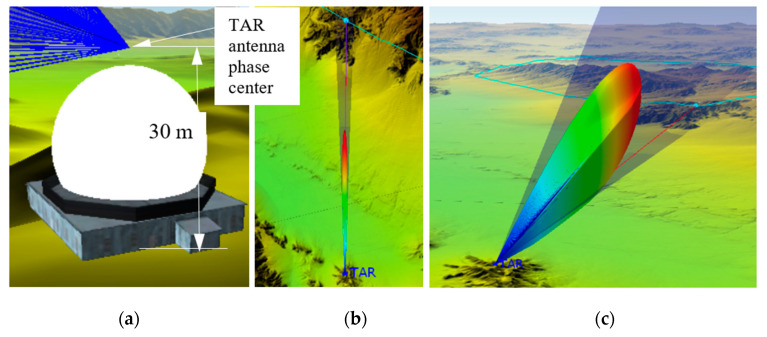
(**a**) TAR antenna phase center location, (**b**) the top view, and (**c**) a 3D view of TAR antenna radiation pattern.

The TAR location was modeled by an STK facility model, which has a white antenna radome shown in Figure 10a. Similar to those RESM antennas, the TAR antenna was also modeled in STK. The TAR antenna phase center and radiation pattern are shown in Figure 10b. The TAR TX parameters are given in Table 4.

### 4.2. The SVE Models of the TAR TX and the 6-Channel RF Digital Receiver

**Figure 11:**
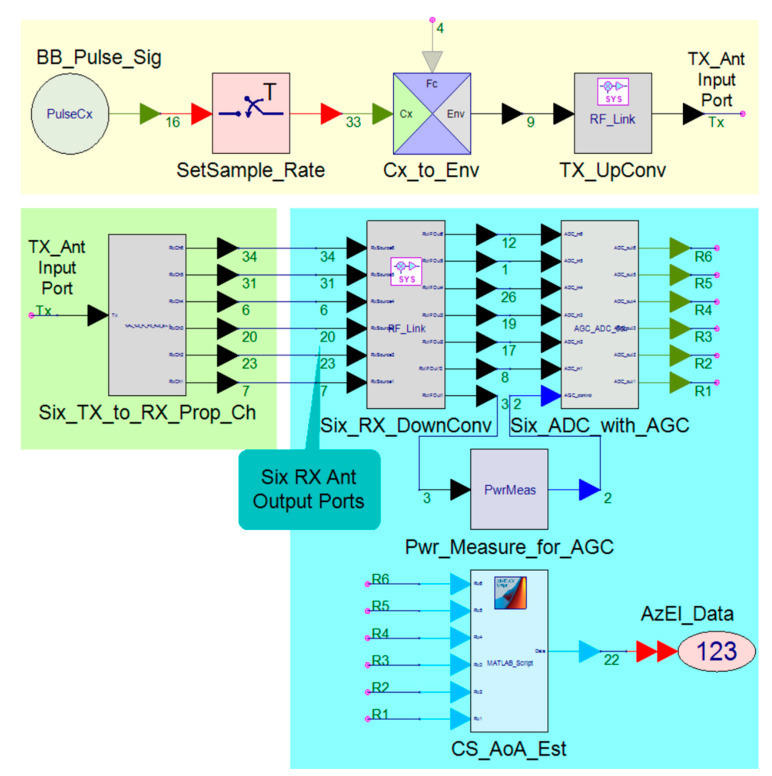
The top-level SVE model for the RESM system to estimate of the TAR signal AoA.

The top-level SVE model is shown in Figure 11. The model includes the RF system models of the TAR TX (yellow box), EM wave propagation channel model (green box), and the RESM RX model with CS-based AoA estimation method (cyan box). These SVE models model signals with complex envelopes at both baseband (BB) and RF carrier, and combine timed synchronous dataflow and dynamic dataflow at the physical layer with RF effects.

The way to use the SVE model in Figure 11 with STK scenario described in the last section is as follows. The TAR TX in the yellow box was in the TAR site modeled in STK, as shown in Figure 10a. The TAR antenna in Figure 10b,c conducts the scanning based on the radar parameters, e.g., sweeping at rate 6 RPM. The 6-channel RESM receiver was installed in the nose of the aircraft. Its 6 CBSAs were inside the radome. The element locations in the antenna RxAnt_xy plane are shown in Figure 9a. The receiving antennas moved with the aircraft based on its flight path given in the scenario. The 3D relationship between the TX and the RX antennas changed dynamically, thus that the antenna patterns changed their pointing directions continuously according to the scenario. The EM wave propagation channel model (green box in Figure 11) responds by fetching the data from STK scenario to the receiver models in SVE at each simulation time step.

Note that, in this study, the main purpose was to study the RESM AoA measurement, and hence, only one system rate was considered. In reality, there could be a number of systems that may all run at their own clock rates without any synchronization. The RF HF-M&S can model each of the systems at its own rate. This is because SVE has the ability to model timed- and untimed systems at their own clock rates [45]. The details of the models in each of the boxes in Figure 11 are given in Appendix B.

## 5. Measurement and RF HF-M&S Results

### 5.1. Lab Measurement Results

**Figure 12:**
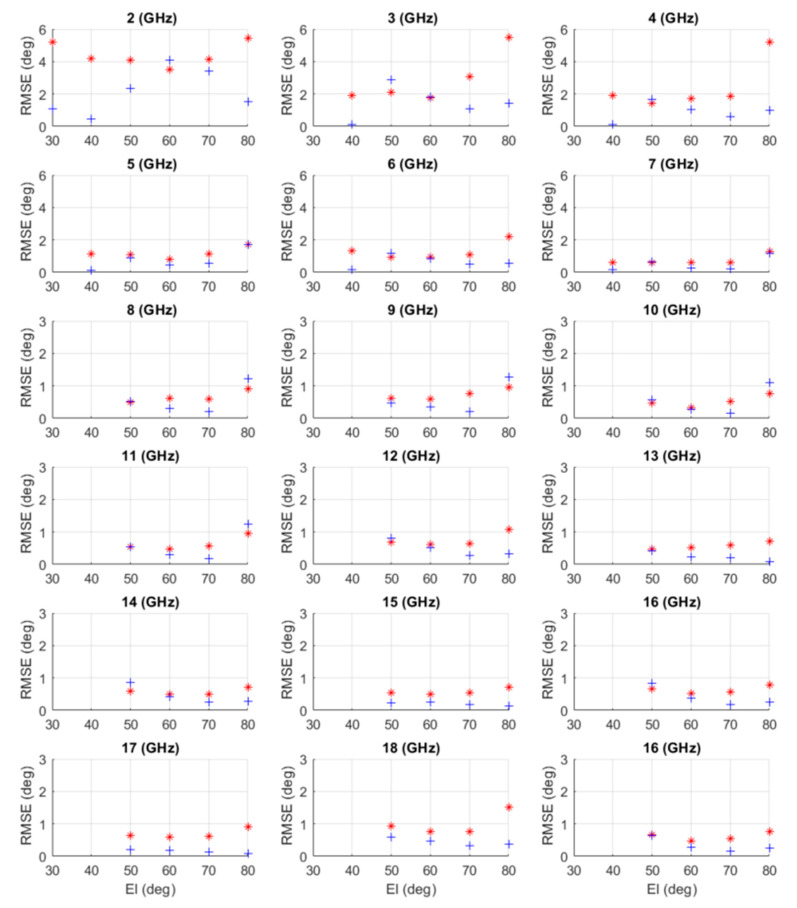
The RMSE of the measured AoA at a different frequency. Red*: RMSE of Az, and blue+: RMSE of El using 13 data points at each El-angle.

The RMSEs of Az- and El-angle estimations from the open lab test are plotted in Figure 12. At each frequency, the RMSEs of Az and El were obtained from 13 Az-directions at each El-angle. The data were removed from RMSE calculations if the estimation error was bigger than 10 (deg) in Az and/or 5 (deg) in El.

From Figure 12, one can find that:

- The AoA measurement system had better AoA estimation when frequency increased from 2 to 8 (GHz). After 8 (GHz), the estimation errors were almost the same. The observation was the same as the results obtained in the M&S in Table 6.
- It shows that El=80 (deg) has bigger estimation errors than that of other El-angles, especially for Az estimations at different frequencies. We believe that this is because of the Az-angles at this level were much closer to each other in the UV-sphere, as shown in Figure 6. Hence, when El-angle was closer to 90 (deg), the algorithm has more challenges to separate Az-angles, and the angular error of the antenna positioner has more influence on AoA measurement results.
- The data also show that the hardware gives better AoA estimations in the El=60 to 70 (deg) range than those of lower El-angles. It is because the problem discussed in the last item is relaxed at these El-angles, and at lower El-angles, the array has a smaller effective aperture, and circular-polarization performance gets worse. One will see later that this effect was not reflected in the M&S, as the perfect circular-polarization was assumed. However, had the measured CBSA circular-polarization data been available, the STK antenna model could have been properly adopted into the M&S.
- Since the CBSA antenna has wider antenna beamwidth at a lower frequency than that at a higher frequency, the measurement results show that:
  ○ AoA measurement can be conducted from 30 to 90 (deg) in El at 2 GHz.
  Up to 7 (GHz), the AoA can be estimated when El reaches 40 (deg). The system cannot give the right measurements at El=30 (deg). Thus data at El=30 (deg) will not be included in the following calculations.
  At higher frequencies, the AoA measurement can only perform El at about 50 (deg), and the system cannot produce any accurate measurements at El=30 and 40 (deg). Hence, the data at El=30 and 40 (deg) will not be included in the following calculations.
- Measurements were repeated at 16 (GHz), and the results had good consistency, as shown in Figure 12.

The RMSEs given in Table 5 are the AoA measurement system overall performance in Az- and El-angles at different frequencies and different IEEE frequency bands. The column of ‘total test/used data’ tells how many data points were supposed to be used in the overall RMSE calculations and how many data points were actually used since at certain measurement points, the estimation error of Az and/or El was bigger than 10 and 5 (deg), respectively, and those data points were removed from the RMSE calculations.

### 5.2. RF HF-M&S Results of the TAR Signal Direction Estimation by the Airborne AoA Measurement System

**Figure 13:**
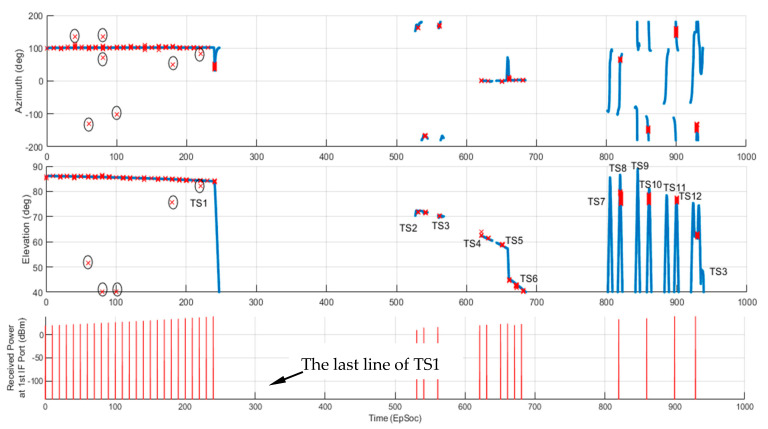
CS—based AoA estimated Az and El (red ‘x’) vs. STK data (blue lines). The details around the last line (in the bottom plot) of the TS1 are given in Figure 15.

**Figure 14:**
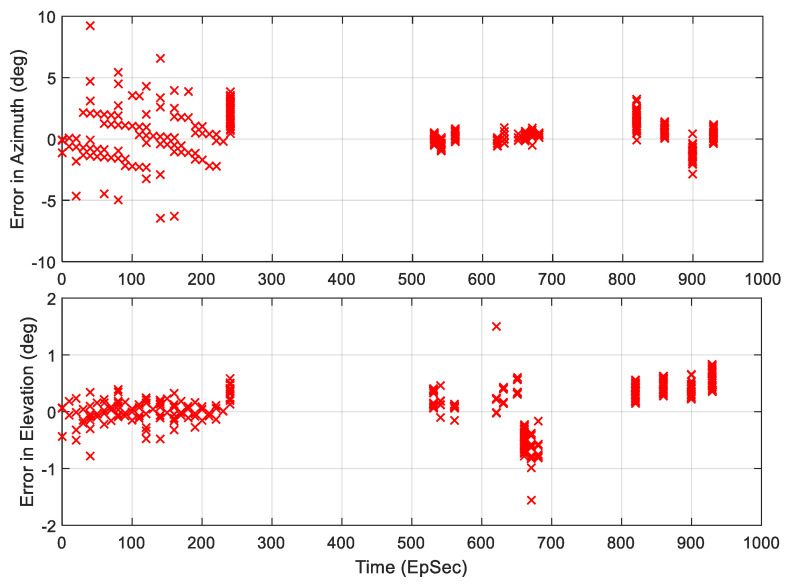
The estimation errors in Az and El.

Figure 13 shows the M&S results of AoA estimation comparison with the ground truth data from STK. As discussed in Section 4, there was a total of 13 TSs within which the TAR signal was within the 3-dB beamwidth of the receiving antennas, and meets LOS condition, i.e., the TAR signal was not blocked by the terrain. The estimation errors for both Az and El are plotted in Figure 14. The RMSEs were 0.95 (deg) and 0.29 (deg) for Az and El, respectively. Note that there were 7 data points (see Figure 13 circled by ellipses) that had big estimation errors, and they were removed from RMSE calculations.

**Figure 15:**
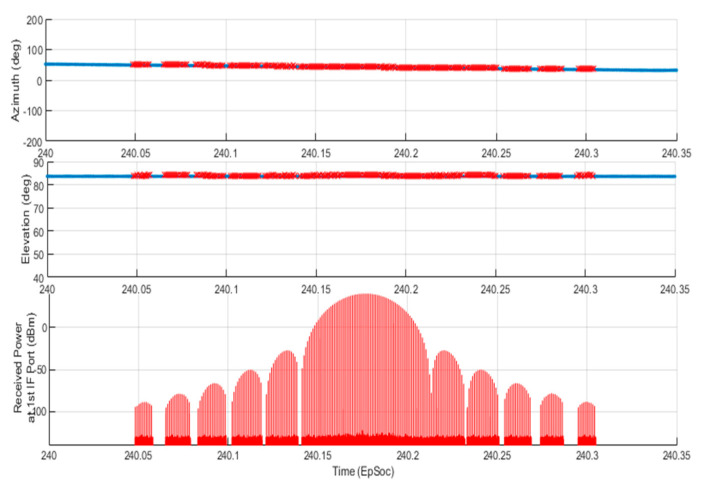
Zoom in plot of the last line of the TS1 in the bottom plot of Figure 13.

The bottom plot of Figure 13 also shows the received signal power level from the first IF output port that is node-3 in the cyan box of Figure 11. Each line in the plot tells the TAR antenna scans over the aircraft in the scenario, and the RESM receiver has the opportunity to conduct AoA measurement. Figure 15 displays the data in between 240 to 240.35 (EpSec), which is the last line of TS1 in the bottom plot of Figure 13.

**Figure 16:**
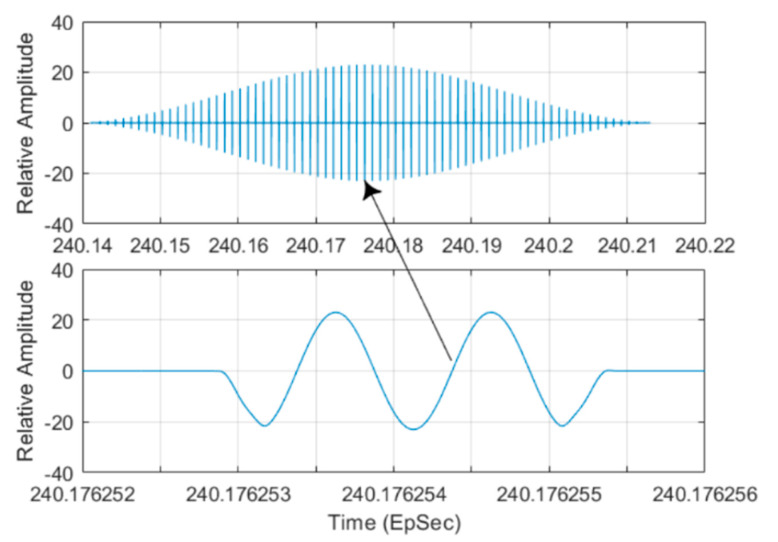
Signal in the main lobe shown in the bottom plot in Figure 15 (**top**), the detailed waveform in the middle line of the top plot (**bottom**).

From Figure 15, one can find that:

- The received signal power level follows the radiation pattern of the TAR antenna when the beam scans through the aircraft.
- The RESM receiver can measure the signal’s AoA even using the TAR antenna side-lobes, since there is only one-way wave propagation from TAR antenna to RESM receiving antennas.
- Although during TAR side-lobe scanning through the aircraft, the IF signal can be as low as about -90 (dBm), the receiver still can estimate TAR signal’s AoA. This is because the IF signal of each channel is processed by the AGC circuit, and 2048-point I/Q data FFT are used in the AoA estimation.
- The received IF power plot is also formed by many lines as shown in the bottom plot of Figure 15. The width of those lines is equal to the PW of the TAR. The time between lines is equal to the radar pulse repetition interval. The main lobe and one of these lines is detailed in Figure 16, which shows the RF HF-M&S is a waveform-level REW M&S.

Although there are a total of 13 LOS TSs (Table 3) that the RESM can measure TAR signal AoA, one can see from Figure 13 that some TSs have no AoA measurements, e.g., TS7 and TS9. This is because the TAR antenna main beam does not have a chance to scan over the aircraft during those TSs.

### 5.3. RF HF-M&S Results of AoA Estimation at Other Frequencies between 2 to 18 (GHz)

Just for demonstration that the AoA measurement system can be used from 2 to 18 (GHz), using the same TAR and aircraft flight in the vignette, AoA estimations at other frequencies were also simulated by properly adjusting TX and RX parameters accordingly for the frequencies listed in Table 6. Since for different frequencies TX and RX EM properties are different, when the aircraft takes the same flight path, the number of AoA calculations were different. The estimation RMSEs are given in Table 6. It can be seen that the system had better estimation capability (1) in El-angle than in Az-angle, and (2) when frequency increased. These observations were consistent with the measured results.

## 6. Conclusions

Using PXIe form-factor digital receiver hardware and 2–18 GHz CBSA elements, an AoA measurement system was developed and tested in an open lab environment. The lab measurement results show that the CS-based 2D AoA measurement system can accurately estimate signal AoA from 2–18 GHz. This article also demonstrates the application of the CS-based 2D AoA method for RESM application through the RF HF-M&S. The M&S and lab test give consistent results with the following observations:

- The CS-based AoA measurement system can be operated from 2 to 18 (GHz);
- The estimation error increases when it operates at lower frequencies;
- The system has better angle estimation in the El-angle than that in the Az-angle;

The overall measured RMSE of estimations are 3.6, 2.74, 1.16, 0.67, and 0.56 (deg) at L, S, C, X, and Ku bands, respectively.

A set of phase-matched CBSA elements will be used in the next version of the randomly-spaced CBSA array. The advanced real-time calibration method will be introduced for the system calibration during the operation, and the system will be installed on an aircraft for the field test.

## Figures and Tables

**Table 1:** Antenna element random locations in the randomly-spaced CBSA array.

Element	X (mm)	Y (mm)
1	0.0	0.0
2	79.96	71.90
3	68.48	−37.65
4	−34.95	−125.35
5	−94.05	−14.55
6	−96.05	99.34

**Table 2:** PXIe modules used in the microwave digital receiver.

#	Module Names	Slot Number on M9018A PXIe Chassis
1	M9037A embedded controller	1
2	M3102A 500MS/s Digitizer	4, 15
3	M9352A Hybrid Amplifier/Attenuator	5, 17
4	M9362A-D01 Quad Down-converter	6–8, 12–14
5	M9300A Frequency Reference Source	9
6	SC5510A 20 GHz Signal Source	11

**Table 3:** Time slots (TS) that TAR inside RESM antenna 3dB beam with LOS.

Time Slot	Start Time (EpSec)	Stop Time (EpSec)	Duration (S)
1	0.000	247.019	247.019
2	526.702	544.506	17.804
3	559.238	567.701	8.463
4	620.557	636.605	16.048
5	642.757	652.396	9.639
6	654.336	684.557	30.221
7	801.536	808.044	6.508
8	815.711	822.204	6.493
9	840.821	847.328	6.507
10	857.084	863.601	6.517
11	882.224	888.674	6.450
12	896.106	902.506	6.400
13	920.115	938.209	18.094

**Table 4:** Parameters of TAR.

**Freq (GHz)**	**TX Power (kW)**	**Pulse Repetition Freq (PRF) (Hz)**	**Pulse Width (PW) (usec)**	**Antenna Gain (dBi)**	**Antenna BW (H/V) (deg)**	**Main Beam Pointing to Sky (deg)**	**Sweep Rate (RPM)**
3.58	700	1000	2	34.77	2/20	10	6

**Table 5:** RMSE of AoA estimations at frequencies and frequency bands.

Freq (GHz)	RMSE in Az (deg)	RMSE in El (deg)	RMSE in Both Angles (deg)	Total Test/Used Data	Overall Performance in Diff. IEEE Frequency Bands (deg)
**2**	4.46	2.44	3.60	78/64	**L-band:** 3.60
3	2.98	1.79	2.46	65/55	**S-band:** 2.74 (avg. of RMSE from 2 to 4 (GHz))
**4**	2.87	1.07	2.16	65/59	
5	1.21	1.00	1.11	65/56	**C-band:** 1.16 (avg. of the RMSE from 4 to 8 (GHz))
6	1.38	0.78	1.12	65/57	
7	0.83	0.67	0.75	65/54	
**8**	0.67	0.68	0.68	52/52	
9	0.75	0.71	0.73	52/52	**X-band:** 0.67 (avg. of the RMSE from 8 to 12 (GHz))
10	0.54	0.64	0.59	52/52	
11	0.66	0.70	0.68	52/52	
**12**	0.78	0.51	0.66	52/50	
13	0.57	0.26	0.45	52/52	**Ku-band:** 0.56 (avg. of the RMSE from 12 to 18 (GHz))
14	0.58	0.51	0.55	52/52	
15	0.59	0.20	0.44	52/50	
16	0.62	0.37	0.51	104/101	
17	0.71	0.16	0.51	52/50	
**18**	1.05	0.43	0.81	52/46	

**Table 6:** RF HF-M&S results of AoA estimation RMSE (Deg) at other frequencies.

Freq (GHz)	Total Number of AoA Points Calculated	Number of Points Est. Error > 10 (deg)	Az and El (without Points Est. Error > 10 (deg))	
2.00	10891	26	2.42	1.42
**3.58**	**8889**	**7**	**0.95**	**0.29**
7.12	6941	4	0.83	0.11
10.13	6152	4	0.88	0.20
13.17	5525	5	0.71	0.10
15.13	5233	7	0.79	0.30
18.00	4655	5	0.77	0.08

Note that, in reality, TARs were hardly operating at high frequencies, as most long range TARs use L- and S-band frequencies. Here we just used M&S to demonstrate the CS-based 2D AoA scheme can be used from 2 to 18 (GHz).

**Figure A1:**
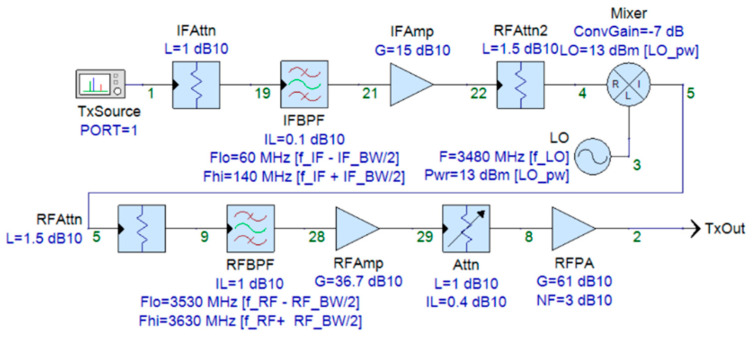
RF up-converter of TAR in TX_UpConv block of Figure 11.

**Figure A2:**
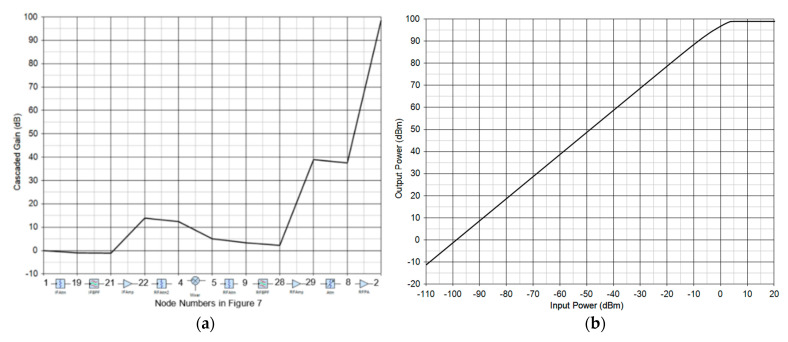

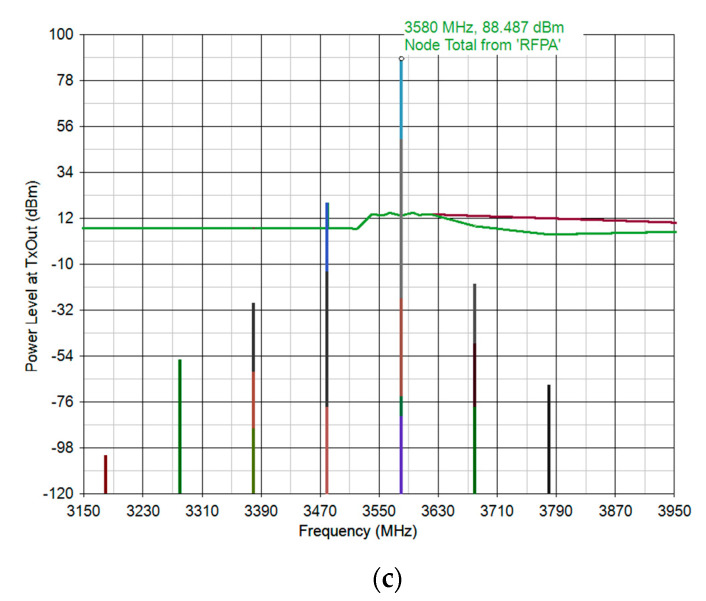
The cascaded gain (**a**), compression curve (**b**), and output power (with input −10 dBm) (**c**) of the TAR RF up-converter section.

**Figure A3:**
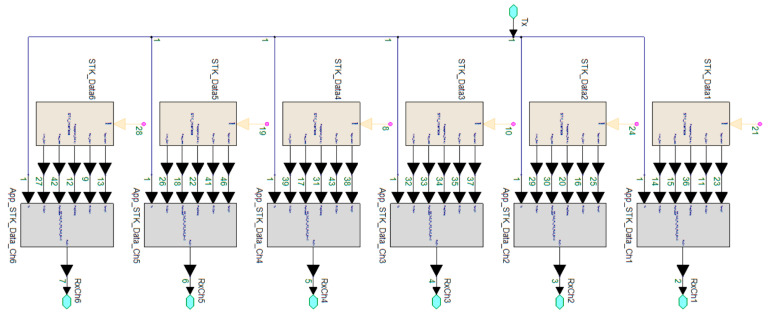
Six EM propagation channel models obtain data from STK to SVE and apply to TAR TX-signal.

**Figure A4:**
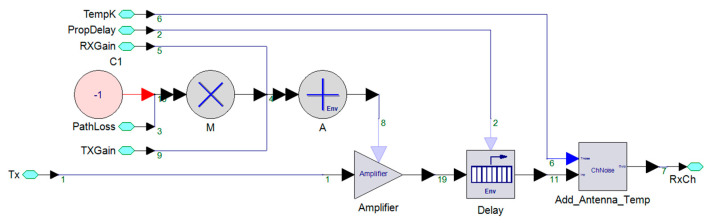
Details inside App_STK_Data model in Figure A3 to apply STK data onto the complex envelope TX—signal in Figure A3.

**Figure A5:**
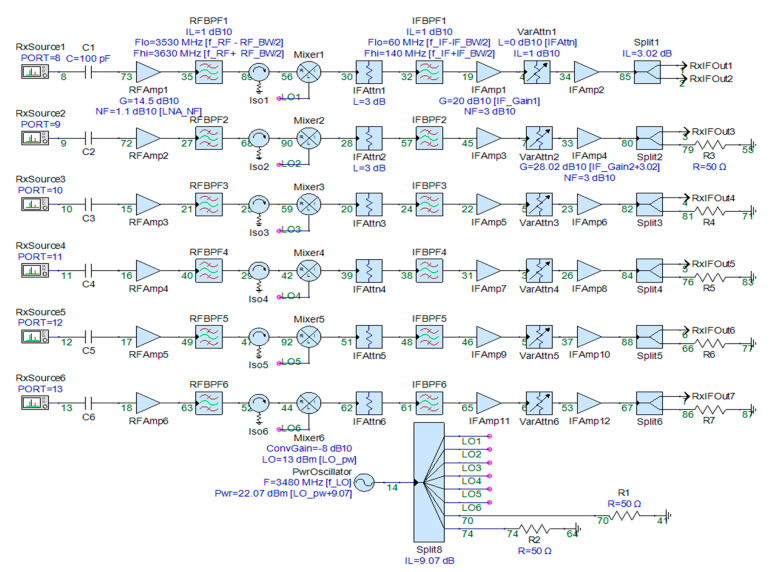
Six RF down-converter model of the RESM receiver.

**Figure A6:**
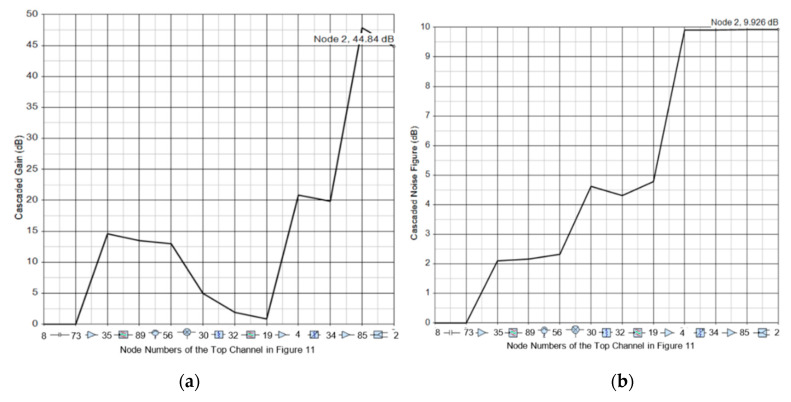

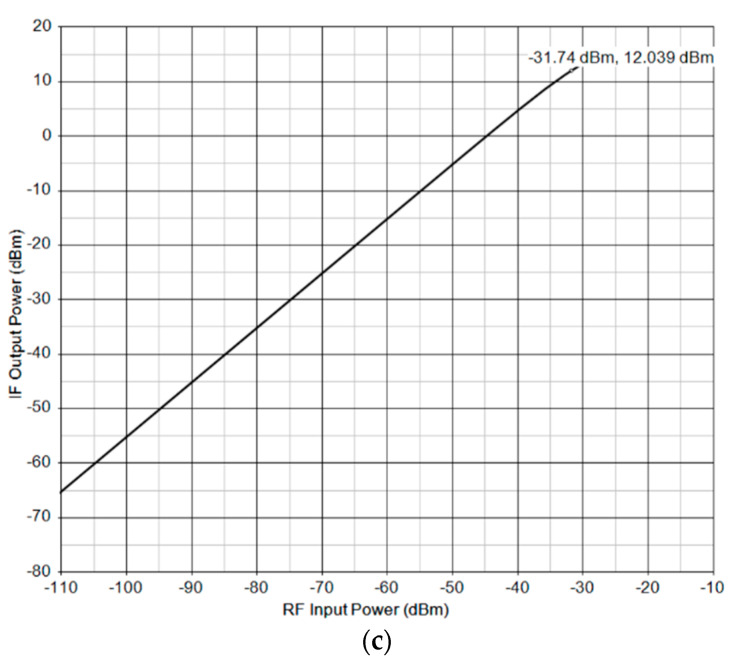
(**a**) The RF down-converter cascaded gain, (**b**) cascaded noise figure, (**c**) compression curve, from RF frequency 3580 MHz to 100 MHz IF frequency.

**Figure A7:**
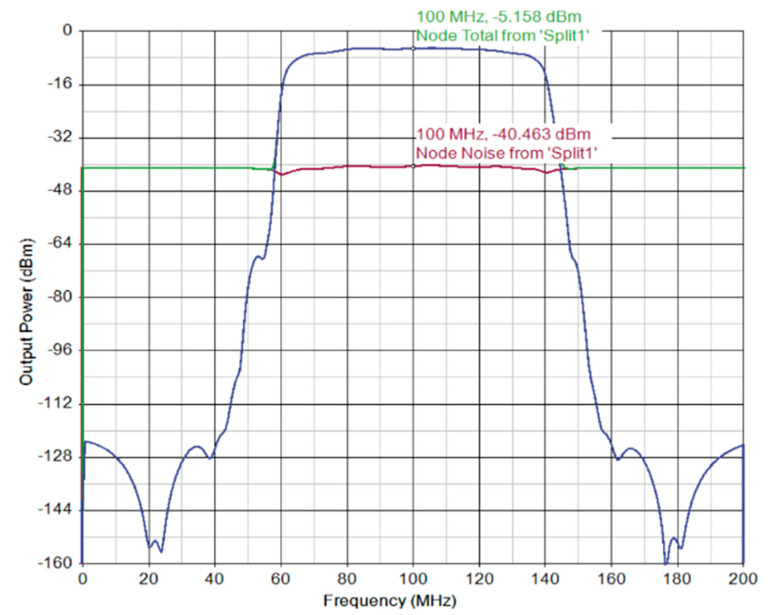
The IF output power of the RF down-converter when sweeping RF signal with an input power level at −50 dBm.

**Figure A8:**
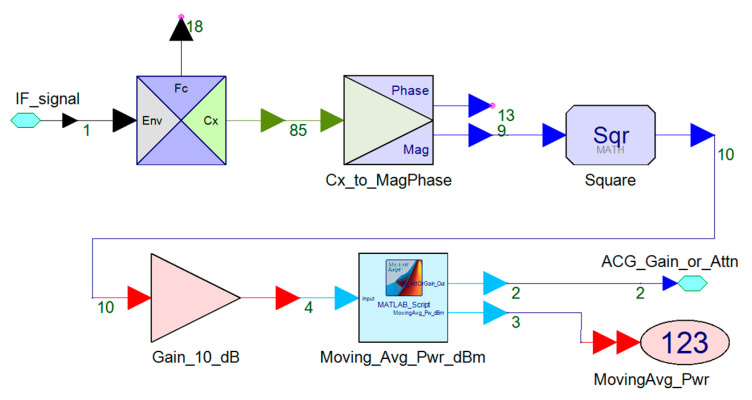
Moving average IF signal power measurement.

**Figure A9:**
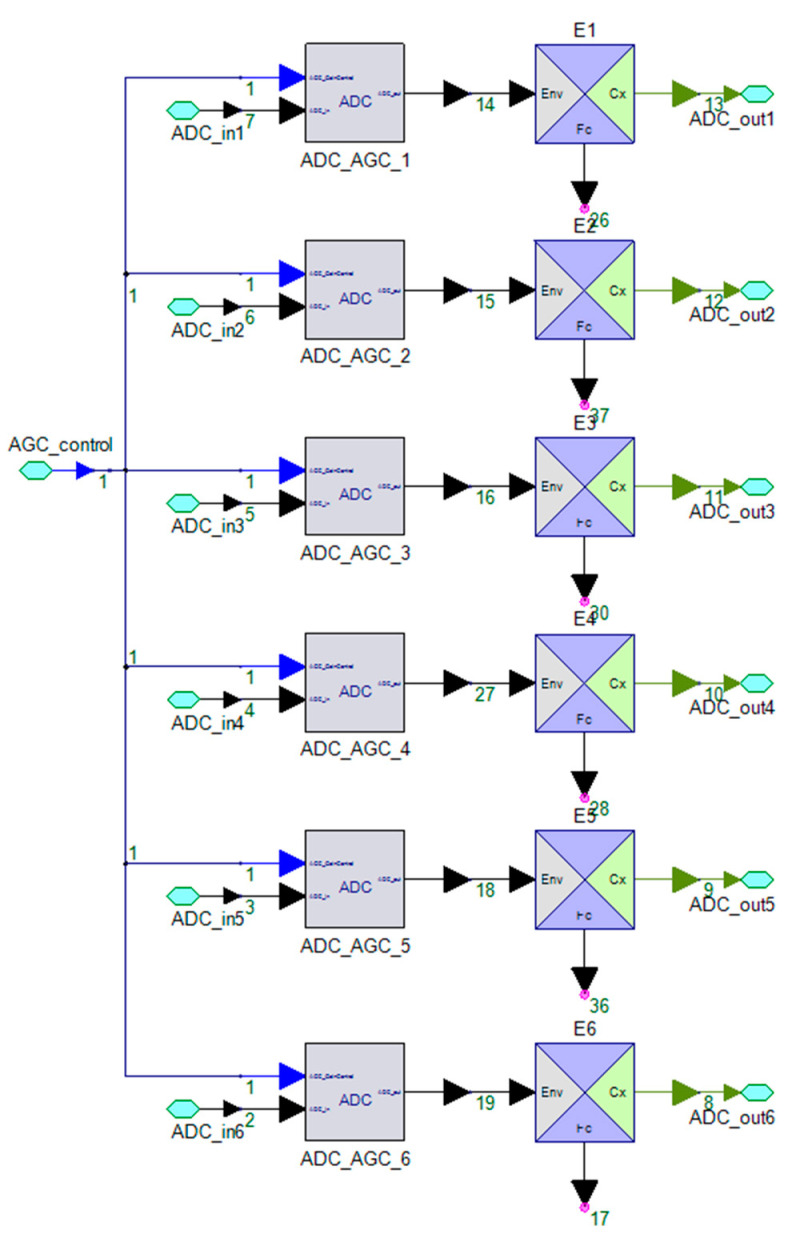
The model inside Six_ADC_with_AGC block.

**Figure A10:**
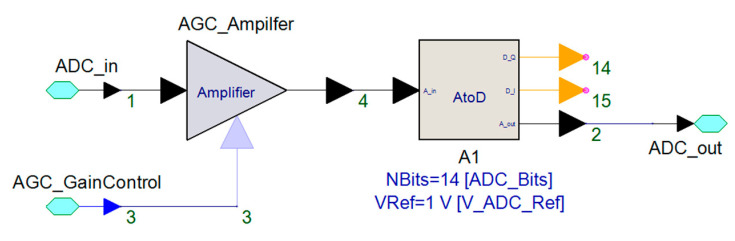
ADC_AGC model.

## Data Availability

All simulated data except the model and measured data except hardware are available to the readers.

## Appendix A. Brief on the RF HF-M&S for REW Engagement Study

The RF HF-M&S is an approach that allows researchers to study REW methods through modeling radar and REW system behaviors at the system level and simulating signals at the waveform level with close to realty REW engagement environment. Different from conventional multi-channel radar signal generation that only produces phase-continuous and phase-coherent (PC-PC) signals at pulse-by-pulse-level, the RF HF-M&S can be used to generate multi-channel PC-PC signals at sample-level in RF and/or intermediate frequency (IF) used by the high-performance signal generators, such as agile signal generator [52] and arbitrary waveform generator [53,54], for REW hardware-in-the-loop tests and studies.

Taking the advantages of different software packages, the RF HF-M&S essentially is a co-simulation of STK, SVE, and Matlab/ Simulink. STK provides a detailed 3D environment and time-based physical and electromagnetic (EM) relationship between any pair of RF Transmitter (TX) and receiver (RX) antennas installed on different platforms, while these platforms move according to the scenario. The RF hardware (not including the antennas) of any TX-RX pair in the scenario is modeled in SVE. This includes RF/microwave systems, signal generation in the TX, digital sampling, and signal processing in the RX (which is modeled in Matlab). The feedback and logic control loops between different parts in TX and/or RX are modeled in SVE as well. The RF TX models in the SVE can be designed/modeled as detailed as possible, such as using measured S- and X-parameters, phase noise data, RF component non-linearity, etc., to generate the signal with the TX-specific signal signature [55,56] to test REW algorithms. Normally, signal processing methods are coded in Matlab/Simulink [57,58,59]. The highlights of this M&S approach are as follows:

- It preserves signals’ fidelity from their generation in TXs to the signal processing in RXs at waveform-level;
- The simulation rates are determined by the nature of different RF/microwave and electronic systems, which normally relate to the signal bandwidths. This means it is a true multi-rate RF M&S;
- It models REW problem in spatial-, time-, and frequency-domains (including Doppler frequency) with consideration of the RF/microwave system impairments acting on the signals, and
- As mentioned earlier, the signal amplitude, phase (propagation delay), and Doppler/frequency are changed at each sample in the simulation. More on this will be shown in Appendix B.

## Appendix B. Detail SVE Models in TAR and 6-Channel RESM Receiver

### Appendix B.1. TAR TX Model

A baseband (BB) pulse signal (BB_Pulse_Sig) model shown in the yellow box in Figure 11 produces a complex-valued pulsed waveform signal that has parameters given in Table 4. The BB signal up-converted to TAR carrier frequency (3.58 GHz) by an RF up-converter (Figure A1) that was designed using SVE RF analog signal model software Spectrasys. Its cascaded gain, compression curve, and total output power at the TxOut port, when input BB signal at −10 dBm, are shown in Figure A2. The total output power at the carrier is about 700 kW. Then, the frequency-domain model in Figure A1 is wrapped by the RF-Link model in SVE, called TX_UpConv in Figure 11, and used in the time-domain SVE dataflow simulation.

### Appendix B.2. Six Sets of EM Wave Propagation Channel Data

Since the RESM receiver has six receiving antennas and RF down-converters, there are six pairs of TX-RX from the TAR antenna to RESM receiving antennas. To take into account the EM wave propagation between TX-RX pairs, there are six EM propagation channels in the Six_TX_to_RX_Prop_Ch (green box in Figure 11), and their details are given in Figure A3. In the figure, the STK_Data models are the STK to SVE interfaces, which obtain the SVE simulation required data from the scenario to the SVE RESM receiver at each SVE simulation time step. More details of the simulation time-step or simulation sample rate will be discussed in the following section. For this RF HF-M&S study, the data from STK to SVE are TX antenna gain (TXGain (dB)), EM wave propagation loss (PathLoss (dB)) and delay (PropDelay (sec)), RX antenna gain (RXGain (dB)) and environment temperature, i.e., RX antenna temperature, (TempK (Kelvin)). Here the PathLoss and PropDelay are calculated from the TAR antenna phase center (shown in Figure 10) to each receiving antenna phase center (shown in Figure 9) by STK. Figure A4 shows how these data are applied to the TX-signal inside the App_STK_Data_Ch models and form six signals that output from RESM antennas in Figure 11.

From Figure A4, one can see that the antenna gains and propagation loss are added together and then applied to the TX-signal by an SVE amplifier model, which determines the received signal amplitudes at the input of an RF receiver, and the propagation delay is applied to the TX-signal by an SVE complex envelope signal delay model, which defines the phase of the signal at the input of an RF receiver.

It is important to note that (1) the SVE complex envelope signal delay model not only applies the time delay to the RF carrier frequency but also to the BB signal waveform, thus that it ensures the PC-PC TX-signal from its source to each RESM receiving antenna output port indicated in Figure 11, and (2) there is no need to obtain aircraft instantaneous radial velocity data, i.e., Doppler information, from the scenario in STK to receivers in SVE. The Doppler information is automatically included in the calculation of the propagation delay at each simulation time-step [58,59].

### Appendix B.3. RESM RX Model

Same as the TAR RF up-converter, the 6 RF analog down-converters in the RESM receiver are also modeled by the Spectrasys shown in Figure A5. In this study, these down-converters are identical. However, in general, they can be modeled individually with slightly different component characteristics. The parameters of down-converters are shown in the figure and in Table A1. The down-converter key performances are shown in Figure A6. One can find that the receiver down-converter has about a total of 45 (dB) cascaded gain, 10 (dB) noise figure (NF), and about 12 (dBm) output 1-dB compression point. The frequency response of the down-converter is shown in Figure A7 when the RF input signal is swept and the input power level is at −50 (dBm). The data at 100 MHz in Figure A7 also imitates the down-converter gain and NF.

**Table A1:** Parmeters Used RF TX and RX Models.

f_IF (MHz)	f_LO (MHz)	IF_BW (MHz)	RF_BW (MHz)	LO_PW (dBm)	IF_ Gain1 (dB)	IF_ Gain2 (dB)	LNA_NF (dB)
100	3480	60	80	13	20	25	2.1

After the RF signal is converted to IF, six IF signals are connected to ADC_AGC models in the Six_ADC_with_AGC block shown in Figure 11. The RxIFOut1 port (node 3 of RF_Link in the cyan box in Figure 11) is monitored by the power measurement circuit inside the Pwr_Measure_for_AGC block in Figure A8, which generates a control signal to change the gain of 6 IF signals inside the AGC_Amplier block as shown in Figure A10. A 4-point moving average IF signal power measurement circuit model is given in Figure A8.

### Appendix B.4. Automatic Gain Control (AGC) and Analog to Digital Converter (ADC)

The model inside the Six_ADC_with_AGC block is shown in Figure A9. The ADC input ports receive the IF analog signals at a sample rate of 50.1 (Mega-Sample/sec)). The digitized data are sent to CS-AoA processing block after SVE complex-envelope signal to complex signal conversion blocks (E1 to E6 in Figure A9).

Figure A10 shows the details of ADC_AGC model in Figure A9. The gain of the AGC_Amplifier is controlled by the AGC_GainControl signal that is the AGC_Gain_or_Attn signal from the power measurement circuit in Figure A8. The AGC circuit ensures the amplitude of the IF signal is as close to ADC reference voltage (VRef) as possible.

Theoretically, the sampling rate used in the simulation can be much lower than 50.1 (Mega-Sample/sec), since the bandwidth of the BB signal is about 0.5 (MHz). In order to ensure there are enough sample points within each radar pulse, 50.1 (Mega-Sample/sec) is selected. Thus, there are about 100 sampled points in each pulse, and the M&S time-step is about 19.96 × 10^−9^ (s). As discussed above, at each time step the STK-SVE interface needs to provide data for SVE simulation. Since the finest time-step in the STK simulation is 0.001 (s), the interpolation is used in the STK-SVE interface to obtain data in between STK simulation time-step. There is a trade-off between simulation accuracy and simulation speed since the smaller SVE time-step needs more data points from STK and longer computer simulation time.

## Appendix C. Acronym List

2D	Two-dimensional
AoA	Angle of Arrival
ADC	Analog to Digital Converter
AGC	Automatic Gain Control
ASV	Array Steering Vector
Az	Azimuth
BB	Base Band
BW	Bandwidth or Beamwidth
CBSA	Cavity Backed Spiral Antenna
CS	Compressive Sensing
DF	Direction Finding
El	Elevation
EM	Electromagnetic
EpSec	Epoch Second
ESPIRT	Estimation of Signal Parameters Via Rotation Invariance Techniques
FFT	Fast Fourier Transform
FOV	Field of View
H/V	Horizontal/Vertical
IF	Intermediate Frequency
IG	Information Geometry
LO	Local Oscillator
LOS	Line of Sight
M&S	Modeling and Simulation
MIMO	Multiple-Input Multiple-Output
MUSIC	Multiple Signal Classification
NF	Noise Figure
PC-PC	Phase-Continuous and Phase-Coherent
PRF	Pulse Repetition Frequency
PW	Pulse Width
PXIe	PCI Express Extension for Instrumentation
RESM	Radar Electronic Support Measures
REW	Radar Electronic Warfare
RF	Radio Frequency
RF HF-M&S	RF High-Fidelity M&S
RMSE	Root Mean Square Error
RPM	Revolutions per Minute
RSA	Randomly Spaced Array
Rx or RX	Receiver
SNR	Signal to Noise Ratio
SOI	Signal of Interest
STK	System Tool Kit
SVE	SystemVue
TAR	Target Tracking Radar
TS	Time Slot
TTR	Target Tracking Radar
Tx or TX	Transmitter
